# Median nerve travel and deformation in the transverse carpal tunnel increases with chuck grip force and deviated wrist position

**DOI:** 10.7717/peerj.11038

**Published:** 2021-03-19

**Authors:** Kaylyn E. Turcotte, Aaron M. Kociolek

**Affiliations:** School of Physical and Health Education, Nipissing University, North Bay, Ontario, Canada

**Keywords:** Ultrasound, Median nerve, Wrist posture, Displacement, Shape, Carpal Tunnel Syndrome

## Abstract

**Background:**

We assessed median nerve travel and deformation concurrently to better understand the influence of occupational risk factors on carpal tunnel dynamics, including forceful chuck gripping and deviated wrist positions.

**Methods:**

Fourteen healthy right-hand dominant participants performed a chuck grip in 6 experimental conditions: two relative force levels (10% and 40% of maximum voluntary effort); three wrist positions (15° radial deviation, 0° neutral, 30° ulnar deviation). Chuck grip forces were measured with a load cell while the transverse cross-section of the carpal tunnel was imaged via ultrasound at the distal wrist crease. Images of the median nerve were analyzed in ImageJ to assess cross-sectional area, circularity, width, and height as well as travel in the anterior-posterior and medial-lateral axes.

**Results:**

We found a main effect of deviated wrist position on both anterior-posterior and medial-lateral travel, with the greatest nerve travel occurring in 30° ulnar deviation. There was also a significant interaction between chuck grip force and deviated wrist position on cross-sectional area. Specifically, the area decreased with 40% vs. 10% chuck grip force when the wrist was in 30° ulnar deviation; however, there were no changes in 0° neutral and 15° radial deviation.

**Discussion:**

Overall, we demonstrated that forceful chuck gripping in deviated wrist positions influenced carpal tunnel dynamics, resulting in both migratory and morphological changes to the median nerve. These changes may, in turn, increase local strain and stress with adjacent structures in the carpal tunnel. Future studies mapping contact stress between structures may further elucidate injury development of work-related carpal tunnel syndrome.

## Introduction

Carpal tunnel syndrome (CTS) is a common disorder in the workplace (Newington, Harris & Walker-Bone, 2015), which causes numbness, pain, and loss of sensitivity in the thumb, index, and long finger (Dahlin et al., 2010). In severe cases, sensorimotor function may be compromised, including decreased accuracy and increased variability of precision grip movement and force (Li et al., 2015; Nataraj et al., 2014). CTS is associated with a number of physical work characteristics, including prolonged exposure to hand-arm vibration, forceful efforts, repetitive movements as well as non-neutral wrist and hand positions (Harris-Adamson et al., 2015; Kozak et al., 2015; Van Rijn et al., 2009). While it is well-established that compression of the median nerve (MN) causes CTS (Aroori & Spence, 2008; Barr, 2004), the underlying mechanisms of injury and particularly how they relate to occupational risk factors are complex and likely multi-factorial (Staal, Bie & Hendriks, 2007).

Two frequently identified injury mechanisms in the development of work-related CTS include elevated hydrostatic pressure and increased contact stress on the MN, both of which contribute to its compression (Keir & Wells, 1999; Luchetti et al., 1989; Vignais, Weresch & Keir, 2016; Viikari-Juntura & Silverstein, 1999). Awkward wrist positions, including wrist flexion-extension and radioulnar deviation as well as loading the fingertips increase carpal tunnel pressures (Rempel, Keir & Bach, 2008; Rempel et al., 1997). Similarly, Kociolek, Tat & Keir (2015) showed that greater tendon load in a flexed wrist position increased frictional work, in part, due to higher contact stress of carpal tunnel structures against the transverse carpal ligament. However, these biomechanical phenomena are not necessarily mutually exclusive and likely interact with each other in the development of work-related CTS (Zhao et al., 2011). One carpal tunnel structure associated with both pathomechanisms is the subsynovial connective tissue (SSCT), a loose collagenous matrix organized in several layers and interconnected by small perpendicular fibrils (Guimberteau et al., 2010). CTS is histologically characterized by fibrosis and thickening of the SSCT thereby increasing volume within the carpal tunnel (Ettema et al., 2006; Tat, Wilson & Keir, 2015), which may increase hydrostatic pressure and contact stress in concert.

While the precise effects of increased pressure and contact stress on carpal tunnel dynamics are not well understood, several studies have used diagnostic imaging to assess travel or deformation patterns of carpal tunnel structures (Cowley et al., 2017; Gabra et al., 2016; Loh & Muraki, 2014; Loh et al., 2018; Lopes et al., 2011; Nanno et al., 2015; Van Doesburg et al., 2011; Van Doesburg et al., 2012; Wang et al., 2014a; Wang et al., 2014b; Yoshii et al., 2009). Loh, Nakashima & Muraki (2016) evaluated the MN in different gripping conditions using ultrasound, and found its cross-sectional area decreased with a clenched fist versus an unclenched fist. Cowley et al. (2017) assessed MN deformation during forceful gripping in different static wrist flexion-extension positions. Although MN cross-sectional area did not change, MN circularity increased in 30° wrist flexion compared to 0°, which was further pronounced with greater grip force. Gabra et al. (2016) examined travel of the flexor digitorum superficialis and profundus tendons in the carpal tunnel during forceful finger pulls and found that flexing the wrist caused the tendons to move palmarly, decreasing the distance between the tendons and the transverse carpal ligament.

Although researchers have assessed travel and deformation of carpal tunnel structures individually, there remains a need to consider both constructs in concert. It is possible that travel patterns of carpal tunnel structures may be related to changes in deformation owing to localized differences in hydrostatic pressures and contact stresses. Furthermore, there remains a need to better understand the effects of occupational risk factors on carpal tunnel dynamics, including forceful efforts and non-neutral wrist/hand positions. The purpose of this study was to examine MN travel and deformation concurrently during forceful chuck gripping with radial and ulnar deviated wrist positions. We hypothesized that a higher chuck grip force would increase travel and circularity of the MN, and this effect would be emphasized in deviated wrist positions.

## Materials & Methods

### Participants

Fourteen healthy right-hand dominant participants (7 men and 7 women) completed this study (Table 1), which was approved by the Nipissing University Research Ethics Board (File #101506). All participants provided written informed consent before completing a health screening questionnaire. Exclusionary criteria included diabetes mellitus, thyroid condition, gout, amyloidosis or sarcoidosis, renal failure or hemodialysis, degenerative joint disease, arthritis of the wrist/hand, corticosteroid injection, Colles fracture, radial malunion, peripheral neuropathy, carpal tunnel syndrome, flexor tendinopathy, wrist/hand surgery or injuries, or symptoms of pain, tingling, or numbness of the wrist/hand in the past year.

**Table 1:** Participant characteristics (*n* = 14).

	**Mean ± SD**
Age (years)	24.21 ± 6.13
Height (cm)	173.79 ± 11.05
Weight (kg)	75.07 ± 13.37
BMI (kg/m^2^)	24.79 ± 3.44

### Experimental setup

**Figure 1:**
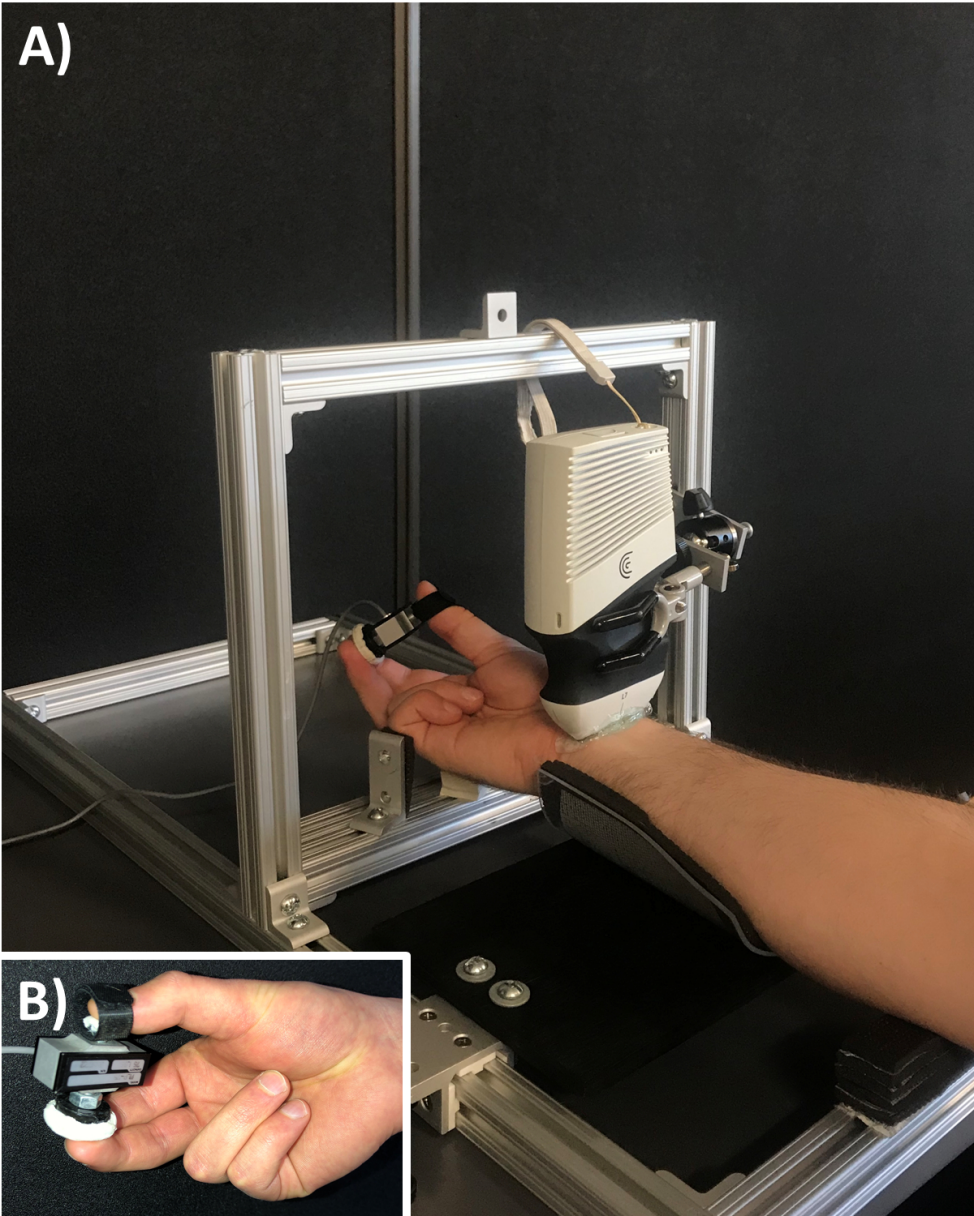
Experimental setup. (A) The custom apparatus was adjustable to support each participant’s anthropometry. The apparatus included supports for the forearm and wrist while a clamp fixed the ultrasound transducer. (B) The load cell consisted of pushbuttons on either side to perform a chuck grip.

Each participant was seated with their right arm supinated in a custom testing apparatus (Fig. 1), which was constructed from slotted aluminum extrusion and linear motion components to provide participant specific adjustability (80/20 Inc., Columbia City, IN). The apparatus included a padded splint to support the forearm. At the level of the metacarpal bones, two adjustable supports fixed the hand to maintain the wrist in either radial or ulnar deviation (depending on the experimental condition). The ultrasound probe was fixed in place with a custom holding device consisting of a locking ball-and-socket joint and three-prong clamp. The probe was positioned parallel to the distal wrist crease, at the base of the thenar eminence with a generous amount of gel to optimize acoustic coupling and minimize contact pressure during ultrasound imaging. Participants interacted with a custom chuck grip dynamometer, which consisted of a single-axis load cell (MLP-75, Transducer Techniques, Temecula, CA) and two pushbuttons attached on each side (1 for the thumb and 1 for the opposing index and middle fingers; 1.13 cm^2^ and 5.73 cm^2^, respectively) with a fixed grip span of 4.5 cm (Fig. 1).

### Experimental protocol

**Figure 2:**
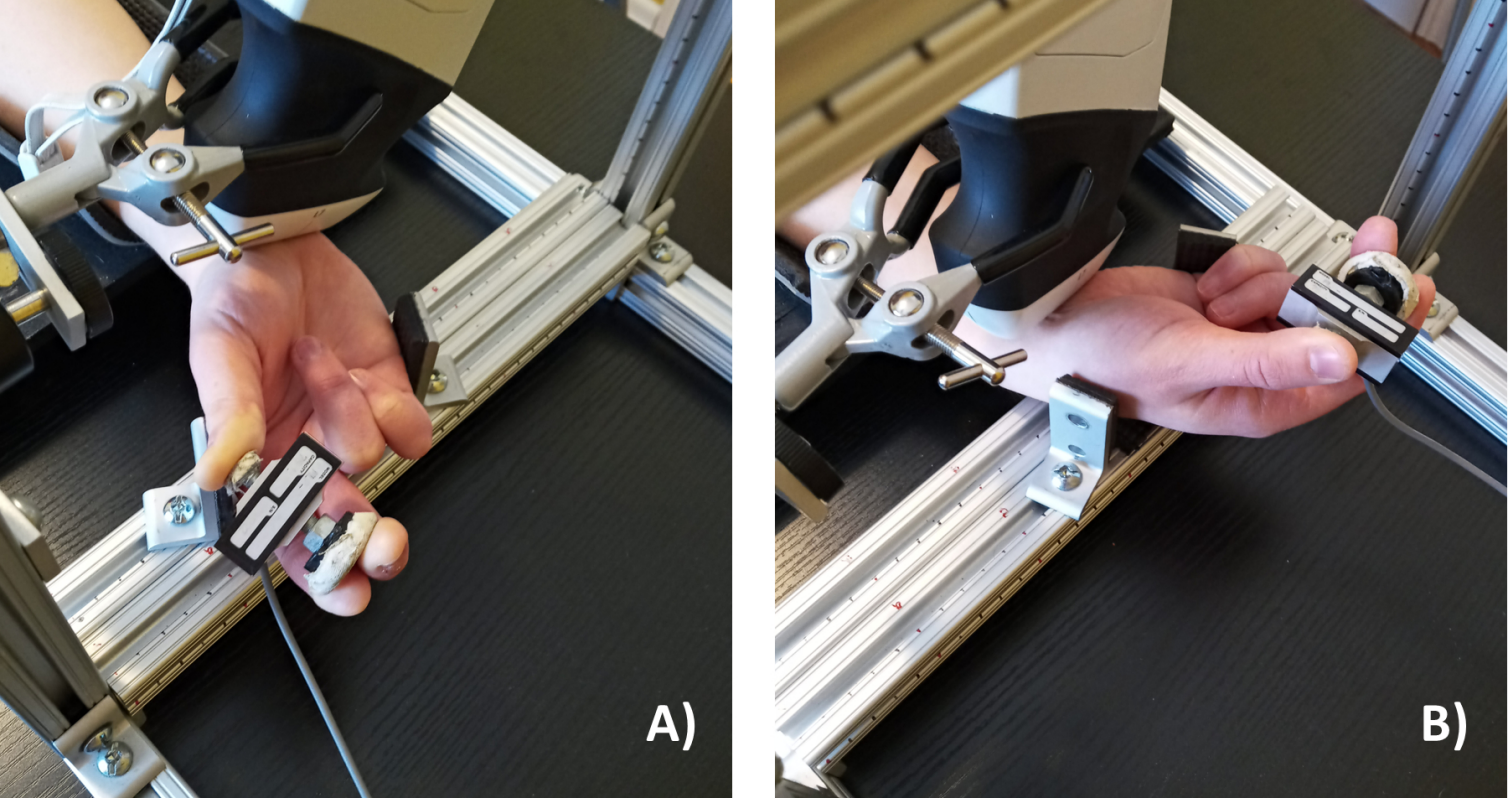
Wrist radioulnar deviation test positions. (A) 15° radial deviation and (B) 30° ulnar deviation tested during the chuck grip task with the forearm supinated. Wrist flexion-extension was maintained in neutral (0°) throughout all test conditions.

Participants were asked to perform a chuck grip, which involved placing the thumb on the smaller push button of the custom dynamometer and placing the index and long fingers on the larger push button of the dynamometer in opposition to the thumb (Fig. 1). Participants performed the chuck grip in both a low force and a high force condition, set relative to their individual strength at 10% and 40% of their maximum voluntary effort (MVE). A chuck grip at low and high force levels was selected since this grip type is well documented within field-based studies in ergonomics (Gerhardsson & Hagberg, 2014; MacDonald & Keir, 2018). Also, previous studies have observed changes when chuck gripping forcefully (25% and 50% compared to 0% MVE) with the wrist in 30° flexion (Cowley et al., 2017). We selected 10% MVE to represent the low force condition since the task required the participants to hold the chuck grip dynamometer and it would have been difficult to maintain the chuck grip with absolutely no force (i.e., 0% MVE). 40% MVE was selected as the forceful condition with careful consideration for the loss of mechanical advantage when the wrist was deviated (Li, 2002; Vanswearingen, 1983). The chuck gripping task was performed in a neutral wrist position (0° neutral) as well as 2 deviated wrist positions, including 15° radial deviation and 30° ulnar deviation (Fig. 2). The deviated wrist positions were confirmed with a handheld goniometer on the palmar side with the forearm in supination. Specifically, the fixed arm of the goniometer lined up in the middle of the forearm with the fulcrum positioned in the middle of the radial and ulnar styloid processes, while the moveable arm was aligned with the third metacarpal to measure the deviated wrist position. The custom supports on the testing apparatus were locked in place to ensure deviated wrist positions were maintained throughout the experimental gripping trials. Since this study focused on investigating the effects of radioulnar deviated wrist positions, the wrist flexion-extension angle was maintained at 0° throughout the testing protocol in all experimental conditions.

### Maximum Voluntary Efforts (MVEs)

Before the experimental trials, participants performed 3 chuck grip MVEs using the grip dynamometer with the forearm supinated and wrist in a neutral position (0° radioulnar deviation; 0° flexion-extension). Participants were provided with real-time force feedback on a visual display monitor and the researchers also gave verbal encouragement to elicit maximal responses. MVEs were held for approximately 3–5 s each; 60 s of rest was provided between each successive MVE to minimize neuromuscular fatigue. Maximum chuck grip force was computed as the mean of ±50 ms surrounding the peak during each trial. An average of all 3 MVEs was computed in order to scale the chuck gripping task during the experimental trials (10% or 40% MVE).

### Chuck gripping trials

**Figure 3:**
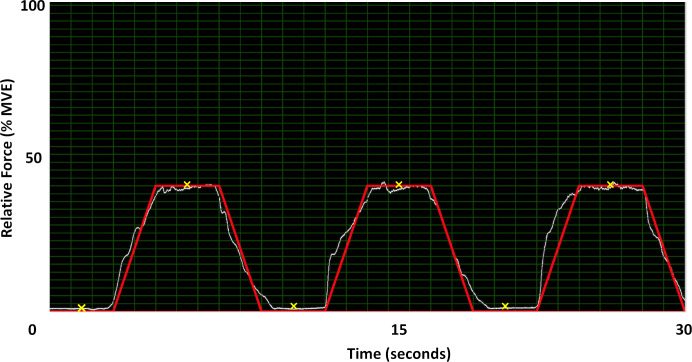
40% MVE force profile in LabVIEW. The *Y*-axis is normalized to 100% of each participant’s MVE while the *X*-axis is time. Participants matched the red line with visual feedback from the load cell (white line). The locations marked with an ‘X’ are where images were extracted for data analysis.

During the experimental chuck gripping trials, participants were asked to complete a force matching task with real-time visual force feedback provided in a custom program (LabVIEW 2014, National Instruments, Austin, TX). Specifically, participants were asked to match a trapezoidal force matching template, which consisted of ramping force up for 2 s, holding the force level (plateau) for 3 s, and ramping force down for 2 s. The force matching template was completed 3 times in succession with 3 s of rest between each trapezoidal profile (Fig. 3). The force ramps were completed at 10% and 40% of MVE within each of the 3 static wrist deviation positions, for a total of 18 static chuck gripping trials (2 chuck grip forces × 3 wrist deviation positions × 3 trials). Prior to the experimental trials, participants were provided with at least 2 practice trials at both force levels with the opportunity for additional practice if needed. The 3 wrist deviation positions were block randomized, and the 2 force levels were counter-balanced within each wrist deviation position to mitigate any potential fatigue or learning effects during the experimental trials.

### Data collection

**Figure 4:**
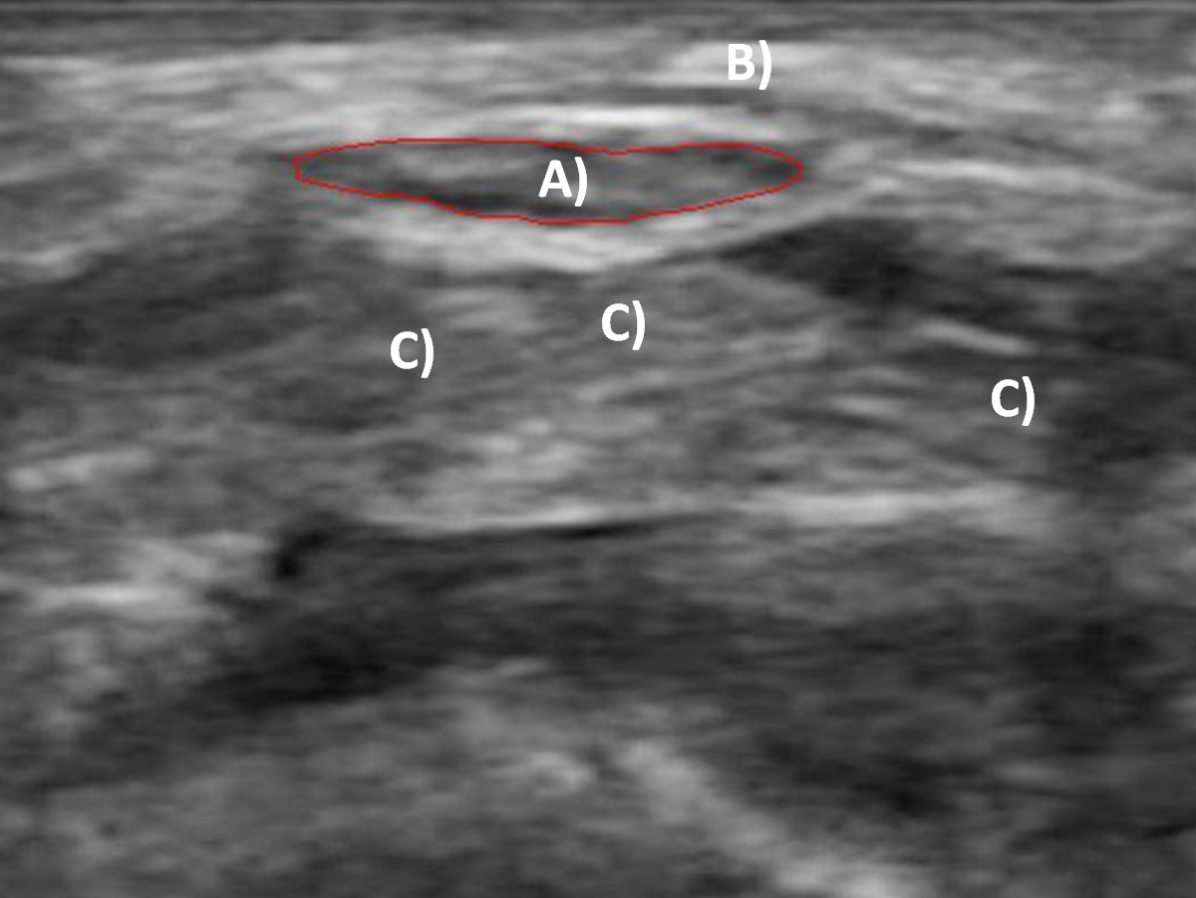
Transverse ultrasound image of the carpal tunnel. (A) Traced median nerve; (B) transverse carpal ligament; (C) flexor tendons.

30-second cine-loops of the transverse carpal tunnel were captured with a linear array ultrasound scanner (L7, Clarius Mobile Health, Burnaby, BC). Carpal tunnel structures identified before collecting each trial included the MN and the lunate carpal bone. The MN was easily identifiable by its hypoechoic area and surrounding hyperechoic boundary near the border of the transverse carpal ligament (Fig. 4). The lunate was located dorsal to the MN and was identifiable by its highly hyperechoic appearance and morphology. All cine-loops were captured in the musculoskeletal (“MSK”) mode to maximize the acquisition frequency of 12 MHz at a scanning depth of 15 mm. Each cine-loop was recorded at 30 frames per second for a total of 900 frames per recording. Both MVE and experimental gripping forces were collected in the custom LabVIEW program. Grip forces were amplified and digitally sampled via a USB high speed analog-to-digital converter at 1,000 Hz (USB-2533, Measurement Computing, Norton, MA). Grip force and ultrasound collections were initiated simultaneously for synchronization.

### Data analysis

Images were extracted from the captured cine-loops at the mid-points of the force plateaus from each set of the 3 successive force ramp profiles performed for each chuck gripping condition, corresponding to time points 6.5, 16.5, and 26.5 s within the 30-second recordings (Fig. 3). Images were also extracted at the mid-points of the rest periods that preceded each chuck gripping ramp (corresponding to time points 1.5, 11.5, and 21.5 s). The extracted static images of the transverse carpal tunnel were imported into ImageJ (1.52a, National Institutes of Health, Bethesda, MD) to analyze travel and deformation of the MN. The polygon selection tool within ImageJ was used to trace the inner hyperechoic border of the MN (Alemán et al., 2008; Filius et al., 2013). The multi-point tool was also used to determine the coordinate of the most anterior peak of the lunate bone. MN deformation was assessed during each gripping trial, which included the medial-lateral (X) width and anterior-posterior (Y) height of the bounding box that enclosed the nerve, cross-sectional area (CSA), and circularity (Eq. (1)):

(1) \begin{eqnarray*}C= \frac{4\pi A}{{P}^{2}} \end{eqnarray*}

where, C is the dimensionless circularity coefficient ranging from 0 to 1 (1 = perfect circle);

A = area (mm^2^);

p = perimeter (mm).

MN position in both the X and Y directions were expressed relative to the lunate bone in order to account for any potential minor shifts of the ultrasound probe on the wrist during the 30-second cine-loop recordings. MN position was defined by the centroid, which is the average of the X and Y coordinates of all pixels in the traced nerve. X and Y travel was then calculated as the difference between the position at the force gripping plateau relative to the preceding rest period. Since we noted that MN displacement patterns varied considerably between participants, the cumulative travel (i.e., absolute displacement) was calculated to describe the movement. For each participant, the 3 trials completed within each experimental condition were averaged together to characterize mean travel and deformation metrics, which were submitted for inferential testing. For completeness, movement patterns (i.e., ulnar versus radial displacement and palmar versus dorsal displacement) were also categorized for each condition in chi-squared tables.

All grip force data were de-biased, calibrated, and smoothed with a low-pass Butterworth filter at 30 Hz in MatLab (R2017a, Mathworks, Natick, MA). Maximum chuck grip forces from the MVE trials were computed as the ± 50 ms surrounding the peak force. The force levels corresponding to the extracted images at each force plateau (i.e., at 6.5, 16.5, and 26.5 s) were also computed to ensure that the participants achieved the 10% and 40% MVE target forces during the chuck gripping trials. As with the ultrasound metrics, the chuck grip forces of the 3 experimental trials performed within each condition were averaged together and submitted to inferential testing.

### Statistical analyses

Two-way repeated measures analysis of variance (ANOVA) statistical models were used to test effects of force level (10% and 40% of MVE) and wrist deviation position (15° radial deviation, 0° neutral, and 30° ulnar deviation) on the grip force and all MN metrics (X width, Y height, cross-sectional area, circularity, X medial-lateral travel, and Y anterior-posterior travel). A separate two-way ANOVA was run for each metric. Significant main effects and interactions were further analyzed with Tukey’s Honest Significant Difference test to control familywise error (alpha set at 0.05).

## Results

### Chuck grip forces during Maximum Voluntary Efforts (MVEs)

The maximum chuck grip force elicited over 3 consecutive MVE trials was a mean (±95% confidence interval) of 94.20 ± 11.47 N (Table 2). The coefficient of variation over the 3 MVE chuck gripping trials was 4.9 ± 1.4%, demonstrating good trial-to-trial repeatability. Participants successfully followed the force level templates during the experimental trials; the average chuck grip force at the midpoint of the plateaus, where ultrasound images were extracted for analysis, for the 10% MVE condition was 10.1 ± 0.2% and for the 40% MVE condition was 39.1% ± 0.4%. Not surprisingly, there was a main effect of force level (*F*_1,13_ = 52009.10, *p* < 0.01), with significantly higher chuck grip forces in the 40% versus 10% MVE experimental trials. However, there was no effect of wrist deviation position on chuck grip forces during the experimental trials (*F*_2,26_ = 1.81, *p* = 0.18).

**Table 2:** Maximum voluntary efforts (MVEs).

**Trial**	**Mean ± 95% CI (N)**
MVE 1	93.96 ± 10.64
MVE 2	95.04 ± 11.92
MVE 3	93.60 ± 12.35
MVE Ave	94.20 ± 11.47

### MN cross-sectional area and deformation during chuck gripping

**Figure 5:**
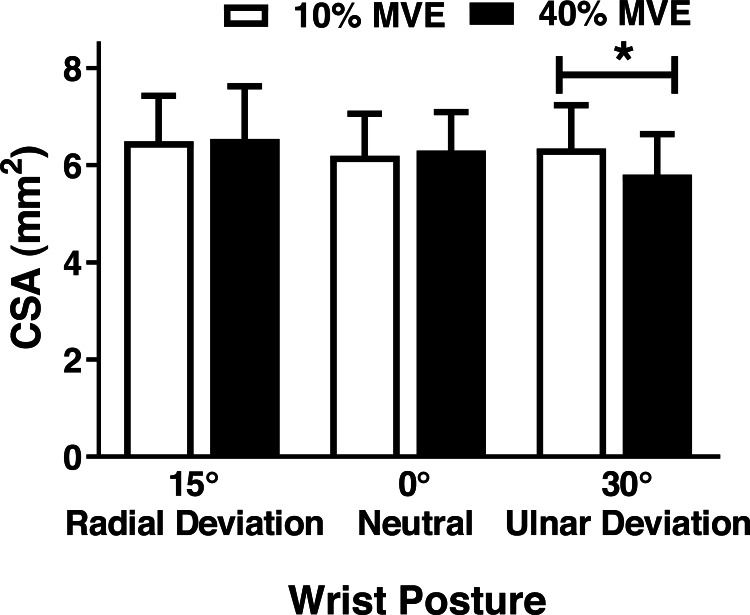
Mean (±95% confidence interval) median nerve CSA (mm^2^) with different chuck grip forces and wrist positions. * represents significant difference.

**Figure 6:**
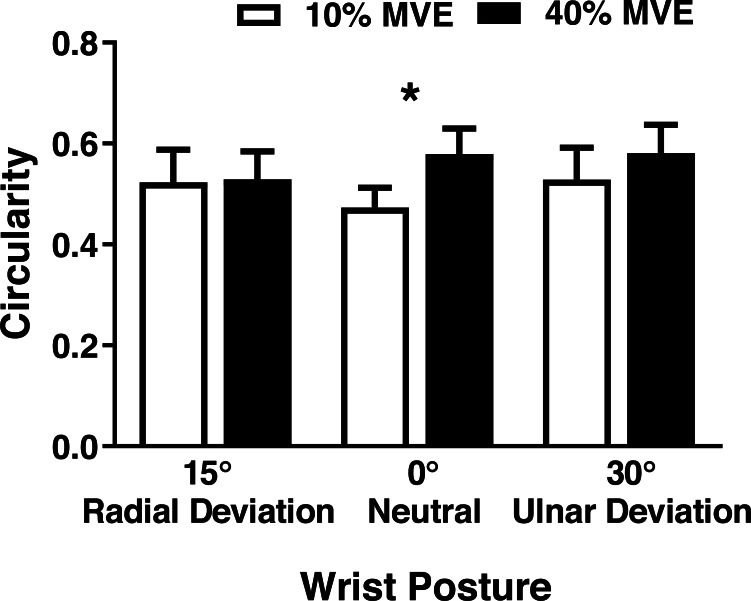
Mean (±95% confidence interval) median nerve circularity (no units) with different chuck grip forces and wrist positions. A value of 1 represents a perfect circle, therefore higher values indicate increased circularity. * represents significant difference.

There was a significant wrist deviation position by force level interaction on MN CSA (*F*_2,26_ = 3.75, *p* = 0.037). CSA was lower in the 40% vs 10% MVE chuck gripping condition when the wrist was in 30° ulnar deviation; however, there were no changes in 0° neutral and 15° radial deviation (Fig. 5; Table 3). We also found a wrist deviation position by force level interaction on circularity of the MN (*F*_2,26_ = 3.77, *p* = 0.04). Circularity increased with greater chuck grip force, but only in the 0° neutral wrist position; while there was a similar trend with chuck grip force in 30° ulnar deviation, it did not achieve significance (Fig. 6). Although there was no significant interaction, there was a main effect of force level on MN width in the medial-lateral axis ( *F*_1,13_ = 15.392, *p* < 0.01). MN width was smaller when chuck gripping at 40% MVE (5.26 ± 0.39 mm) compared to 10% MVE (5.65 ± 0.43 mm). Conversely, there were no significant differences on height of the MN in the anterior-posterior axis.

### MN travel during chuck gripping

**Figure 7:**
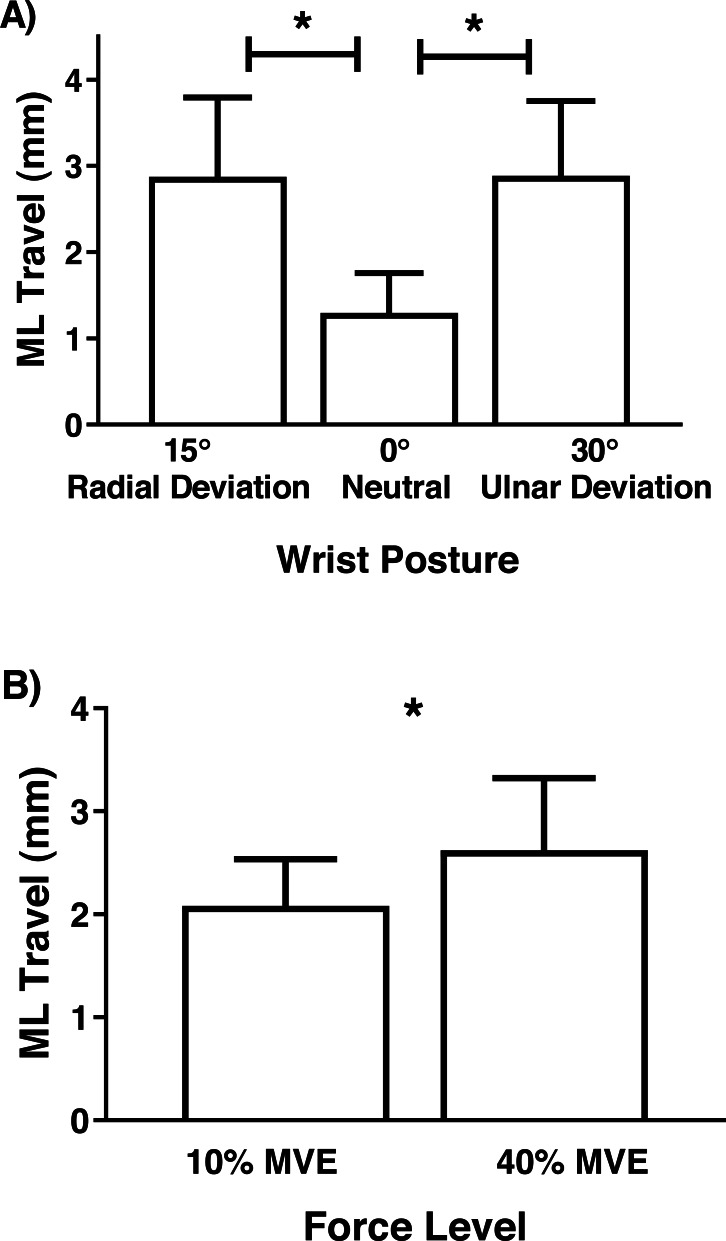
Mean (±95% confidence interval) median nerve travel (mm) in the medial-lateral (ML) axis with different (A) wrist positions and (B) force levels during chuck gripping. * represents significant difference.

**Figure 8:**
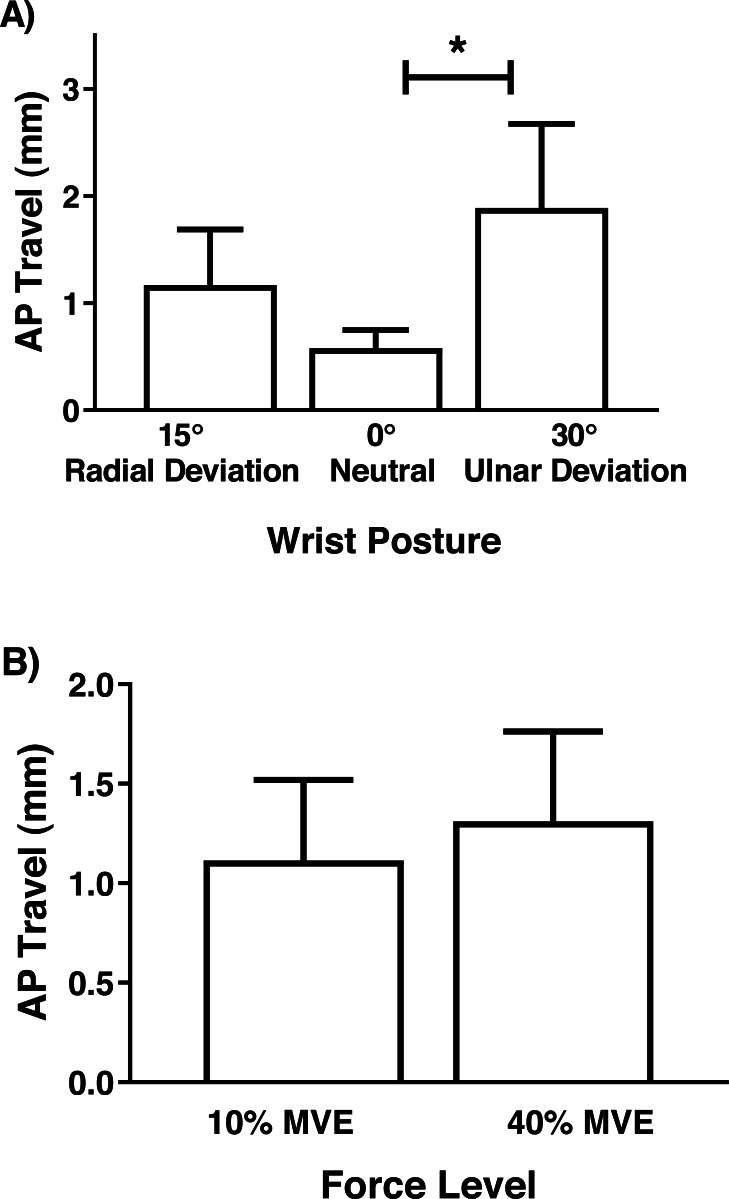
Mean (±95% confidence interval) median nerve travel (mm) in the anterior-posterior (AP) axis with different (A) wrist positions and (B) force levels during chuck gripping. * represents significant difference.

Although there were no significant interactions involving MN travel, there was a main effect of wrist deviation position in the medial-lateral axis (*F*_2,26_ = 6.81, *p* = 0.004), with increased travel in 30° ulnar and 15° radial deviation than 0° neutral (Fig. 7). There was also a main effect of force level (*F*_1,13_ = 4.75, *p* = 0.048), due to increased travel in 40% MVE compared to 10% MVE (Fig. 7). In the anterior-posterior axis, there was only a main effect of wrist deviation position on MN travel (*F*_2,26_ = 8.37, *p* = 0.002). Greater MN travel occurred in 30° ulnar deviation relative to 15° radial deviation and 0° neutral (Fig. 8).

**Table 3:** Mean ±95% CI median nerve deformation and travel in the medial-lateral (ML) and anterior-posterior (AP) axes (*n* = 14).

Wrist Position	%MVE	CSA (mm^2^)	Circularity	Width (mm)	Height (mm)	ML Travel (mm)	AP Travel (mm)
Radial 15°	10%	6.49 ± 0.92	0.52 ± 0.06	5.66 ± 0.69	1.79 ± 0.27	3.00 ± 0.74	1.00 ± 0.57
	40%	6.54 ± 1.08	0.53 ± 0.04	5.63 ± 0.55	1.75 ± 0.22	2.75 ± 1.25	1.33 ± 0.57
Neutral 0°	10%	6.19 ± 0.86	0.47 ± 0.04	5.81 ± 0.51	1.65 ± 0.18	0.59 ± 0.27	0.40 ± 0.16
	40%	6.30 ± 0.80	0.58 ± 0.06	5.19 ± 0.49	1.72 ± 0.14	2.00 ± 0.90	0.76 ± 0.27
Ulnar 30°	10%	6.34 ± 0.90	0.53 ± 0.06	5.48 ± 0.45	1.64 ± 0.20	2.65 ± 0.94	1.94 ± 0.69
	40%	5.81 ± 0.84	0.58 ± 0.06	4.94 ± 0.51	1.79 ± 0.22	3.11 ± 1.00	1.84 ± 0.92

## Discussion

MN travel and deformation were assessed simultaneously during forceful chuck gripping in both neutral and deviated wrist positions, with the underlying goal of exploring the link between dynamic and morphological changes in response to physical risk factors. Overall, we found increased MN travel in both the anterior-posterior and medial-lateral axes while chuck gripping in wrist ulnar deviation. Deformation changes were also observed in an ulnarly deviated wrist position, with a decrease in MN CSA during the 40% versus 10% MVE chuck grip condition. Since the carpal tunnel is a confined area with well-defined borders and multiple structures passing through it, including active structures (e.g., flexor tendons) and passive structures (e.g., MN), the likelihood of the MN becoming deformed against adjacent structures is high. Our study demonstrated that active tension of the flexor tendons during forceful chuck gripping tasks displaced the MN within the carpal tunnel. MN displacement may, in turn, increase local strain and stress with adjacent structures, further elucidating a plausible injury mechanism in the development of work-related CTS.

While it is well-established that CTS results from entrapment of the MN, several different injury pathways are likely involved, including localized contact stress with carpal tunnel borders (Armstrong & Chaffin, 1979; Moore, Wells & Ranney, 1991), strain of surrounding connective tissue due to differential movement between the tendons and MN (Ugbolue et al., 2005; Tat, Kociolek & Keir, 2013; Tat, Wilson & Keir, 2015), and elevated carpal tunnel pressure (Lundborg et al., 1982). These injury mechanisms are often discussed in isolation; however, they are not necessarily mutually exclusive from each other (Zhao et al., 2011). For example, tasks involving forceful pinching and gripping increase pressure within the carpal tunnel, leading to compression of the MN (Goss & Agee, 2010). Moreover, similar tasks involving forceful efforts in flexed wrist positions increase contact stresses resulting from movement of the flexor tendons (Keir & Wells, 1999). These coincident findings demonstrate the need to measure travel and deformation of the MN concurrently using non-invasive techniques such as ultrasound to further elucidate injury mechanisms.

We showed that deviated wrist position interacted with force level during chuck gripping to influence circularity of the MN. Previous studies assessing various physical risk factors for CTS have found similar results (Cowley et al., 2017; Marquardt, Gabra & Li, 2015; Van Doesburg et al., 2011; Wang et al., 2014b; Yoshii et al., 2009). Cowley et al. (2017) examined median nerve circularity during different gripping tasks in wrist flexion-extension positions. They found that MN circularity increased from 0.57 ± 0.05 with no force in a neutral (0°) wrist position to 0.65 ± 0.05 when performing a chuck grip at 50% MVE in 30° wrist flexion. Similarly, Van Doesburg et al. (2011) noted that circularity increased with finger position. Applying an external compressive force at the wrist was also found to increase circularity of the median nerve (Marquardt, Gabra & Li, 2015). Altogether, these findings suggest that tasks involving physical risk factors, especially when occurring in combination, may lead to localized MN strain and contact stress against adjacent carpal tunnel structures.

Previous studies assessing changes in MN CSA have found conflicting results (Cowley et al., 2017; Filius et al., 2015; Lai, Chiu & Law, 2014; Loh, Nakashima & Muraki, 2016; Loh et al., 2018; Mogk & Keir, 2008; Van Doesburg et al., 2010). Cowley et al. (2017) did not find any significant differences in MN CSA from manipulating grip types (4-finger, chuck, and key grips), force levels, or wrist flexion-extension positions. In contrast, Loh et al. (2018) found a significant interaction between finger posture and wrist flexion-extension position, with CSA decreasing when the wrist was flexed or extended at different finger postures. Another study by Loh & Muraki (2014) found significant MN CSA changes involving wrist flexion and extension; however, there were no differences between radial versus ulnar deviation. In our study, we observed a decrease in MN CSA in ulnar deviation, but only when chuck gripping at a high force level of 40% MVE. Differences in force levels may, in part, explain these discrepancies across studies.

Increased MN travel in flexed or extended versus neutral wrist/finger positions have been previously documented using ultrasound (Van Doesburg et al., 2012; Wang et al., 2014a; Wang et al., 2014b; Yoshii et al., 2009). For example, during a finger flexion task, Yoshii et al. (2009) found an average of 2.3 mm and 0.25 mm displacement in the radial and palmar direction, respectively. Van Doesburg et al. (2012) found MN displacement in the ulnar direction ranging from 0.49 mm (index finger motion) to 1.40 mm (full-fist motion), both in healthy participants. While these values are smaller than our study (0.59 mm to 3.11 mm in the medial-lateral axis), we calculated absolute travel distances (instead of displacements) since movement patterns varied between participants. In addition, by design, our study involved relatively high force levels during chuck gripping, which may increase the interaction between the MN and the surrounding tendons as they develop active tension to support forceful gripping. We did not analyze the flexor tendons specifically; however, anecdotally, tendon movement was apparent during collection and analysis. When the tendons develop active force, they displace palmarly, which results in travel of MN thereby mitigating compressive forces to some extent (Wang et al., 2014b). Since we did not measure the adjacent tendons in our study, we cannot definitively elucidate the precise causes for the travel of the MN, which is a passive structure in the carpal tunnel. Future studies may benefit from investigating relative displacements between the MN and flexor tendons.

Interestingly, we noticed a large variation in MN movement patterns when looking at its direction of travel (Table S1). While a greater percentage of participants demonstrated MN travel in the radial and palmar directions when the wrist was ulnarly deviated, and ulnar and palmar directions when the wrist was radially deviated, large variability in the direction of travel may warrant further investigation in future studies. Goetz et al. (2010) used magnetic resonance imaging to assess location of the MN and found significant variation in MN location from day-to-day. This may explain why we noticed such inconsistency in movement patterns between our participants. However, we do think this variability would be an interesting point to investigate further, perhaps assessing movement patterns in individuals with CTS.

This study is not without limitations. All ultrasound scans at the distal wrist crease were performed with the forearm in supination to optimize acoustic coupling, which may have generated additional strain on the extrinsic finger flexors. However, previous MRI studies performed with the forearm in a prone position provided similar images and results (Goetz et al., 2013; Nadar, Dashti & Cherian, 2013). While our results infer greater localized strain and/or stress on the MN during forceful chuck gripping, we only measured travel and deformation metrics. Future elastography studies could provide a direct assessment of strain/stress. Furthermore, due to the manual nature of image analysis, results are dependent on the images and the user performing the analysis. Junck et al. (2015) previously demonstrated high intra- and inter-rater reliability using ultrasound to assess MN morphology. MN metrics in our investigation corresponded with previous studies assessing validity and reliability of ultrasound measurement (Alemán et al., 2008; Bueno-Gracia et al., 2017; Impink et al., 2010; Tagliafico & Martinoli, 2013). In addition, all ultrasound images were analyzed by the first author to ensure internal consistency. Finally, this study was only performed on a healthy population. We focused specifically on physical risk factors associated with CTS. If this study were replicated with individuals diagnosed with CTS, it would provide further insight on characteristics of the MN that are directly associated with the disease pathology.

## Conclusions

This study demonstrated the effects of low versus high chuck grip forces when combined with deviated wrist positions on both travel and deformation of the MN. Loading the fingertips with a high force level (40% MVE) and an ulnar deviated wrist position caused a decrease in MN CSA. Furthermore, it was found that radial and ulnar deviation caused significant travel of the MN. While it is clear that median nerve travel and deformation occurred in concert, the implications with respect to injury development need to be further explored. Future studies mapping changes in contact stress may provide us with further information on the precise injury pathway of CTS.

##  Supplemental Information

10.7717/peerj.11038/supp-1 Table S1 Median nerve travel including palmar-dorsal and radial-ulnar movement patterns described as a percentage of all participants (*n* = 14)

10.7717/peerj.11038/supp-2 Supplemental Information 2 Median nerve cross-sectional area (mm^2^) dataset

10.7717/peerj.11038/supp-3 Supplemental Information 3 Median nerve circularity (no units) dataset

10.7717/peerj.11038/supp-4 Supplemental Information 4 Median nerve width (mm) dataset

10.7717/peerj.11038/supp-5 Supplemental Information 5 Median nerve height (mm) dataset

10.7717/peerj.11038/supp-6 Supplemental Information 6 Median nerve medial-lateral travel (mm) dataset

10.7717/peerj.11038/supp-7 Supplemental Information 7 Median nerve anterior-posterior travel (mm) dataset

10.7717/peerj.11038/supp-8 Supplemental Information 8 Participant health screening questionnaire

## Additional Information and Declarations

### Data Availability

The following information was supplied regarding data availability: Raw data is available in the Supplemental Files.
