# Supplementary material for: Median nerve travel and deformation in the transverse carpal tunnel increases with chuck grip force and deviated wrist position
Source: PeerJ. 2021 Mar 19;9:e11038. doi: 10.7717/peerj.11038 (PMC7983861; doi:10.7717/peerj.11038)
Supplement: Table S1 [file peerj-09-11038-s001.docx]

| Condition |  | 15° Radial | | 0° Neutral | | 30° Ulnar | |
| --- | --- | --- | --- | --- | --- | --- | --- |
|  | Direction | Radial | Ulnar | Radial | Ulnar | Radial | Ulnar |
| 10% MVE | Palmar | 21% | 43% | 21% | 14% | 43% | 21% |
|  | Dorsal | 7% | 29% | 14% | 50% | 21% | 14% |
| 40% MVE | Palmar | 0% | 64% | 29% | 36% | 43% | 21% |
|  | Dorsal | 21% | 14% | 14% | 21% | 21% | 14% |
