# Supplementary material for: Median nerve travel and deformation in the transverse carpal tunnel increases with chuck grip force and deviated wrist position
Source: PeerJ. 2021 Mar 19;9:e11038. doi: 10.7717/peerj.11038 (PMC7983861; doi:10.7717/peerj.11038)
Supplement: Supplemental Information 8 [file peerj-09-11038-s008.pdf]

NIPISSING UNIVERSITY

Participant Questionnaire:

The Role of Musculoskeletal Interactions on Wrist and Hand Biomechanics

---

A. PARTICIPANT IDENTIFIACTION (to be completed by the researcher)

Participant #: ________________________ ***Date:*** ________________________

B. DEMOGRAPHICS / ANTHROPOMETRICS

Gender: ________________________

Age: ________________________

Height: ________________________

Weight: ________________________

C. HANDEDNESS

1) Are you are you right-handed, left-handed or ambidextrous?

right-handed ☐ ***left-handed*** ☐ ***ambidextrous*** ☐

D. HEALTH HISTORY

1) Have you ever had any of the following health conditions and/or treatment protocols performed on you currently and/or in the past? [*please check all that apply*]

☐ Diabetes mellitus
☐ Thyroid condition (e.g. Hypothyroidism)
☐ Gout
☐ Amyloidosis or Sarcoidosis
☐ Renal failure or Hemodialysis
☐ Degenerative joint disease
☐ Arthritis of the wrist/hand
☐ Corticosteroid injection
☐ Radial malunion
☐ Colles fracture
☐ Peripheral neuropathy
☐ Carpal tunnel syndrome
☐ Flexor tendinopathy
☐ Wrist/hand surgery
☐ Hand pain/tingling/numbness
☐ Other wrist/hand injury or disorder (not listed above):

____

2) Are you currently on any medications? ***Yes*** ☐ ***No*** ☐

** If ***yes***, please list:

____

____

E. WORK HISTORY

1) Occupation: ____

2) Location of work: ____

3) Hours at work per week: ____

4) Years at current job: ____

5) Typical tasks performed at work:

____

____

____

____

____

6) Have you ever-experienced a work-related wrist or hand injury? ***Yes*** ☐ ***No*** ☐

** If ***yes***, please elaborate (type of injury, onset, symptoms, and treatment):

____

____

____

____

F. MUSIC HISTORY

1) Do you play musical instrument(s)? Yes ☐ No ☐

*** If yes, please answer the following questions:

a) Type of instrument(s):

____

b) Hour per week spent playing instrument(s):

____

c) Self-reported skill level (e.g. hobby, amateur, professional):

____

*** End ***
